# Rotational Characterization of Four-Ring Polycyclic Aromatic Hydrocarbons: Toward the Detection of Fluoranthene and Cyanofluoranthene in Space

**DOI:** 10.1021/acs.jpclett.6c00413

**Published:** 2026-04-17

**Authors:** Daniel Villar-Castro, Carlos Cabezas, Amanda L. Steber, José R. Morán, Selene de la Fuente, Farha S. Hussain, Dolores Pérez, Alberto Lesarri, José Cernicharo, Cristóbal Pérez, Isabel Peña

**Affiliations:** † Centro Singular de Investigación en Química Biolóxica e Materiais Moleculares (CiQUS) and Departamento de Química Orgánica, 16780 Universidade de Santiago de Compostela, 15782 Santiago de Compostela, Spain; ‡ Instituto de Física Fundamental CSIC, C/ Serrano 123, 28006 Madrid, Spain; § Departamento de Química Física y Química Inorgánica, Facultad de Ciencias. - I.U. CINQUIMA, 16782 Universidad de Valladolid, 47011 Valladolid, Spain

## Abstract

The combined use of laboratory rotational spectroscopy and radio astronomical observations remains the most effective approach for identifying molecules in the interstellar medium (ISM). Following the recent detections of several polycyclic aromatic hydrocarbons (PAHs) and their cyano derivatives in the dense Taurus Molecular Cloud (TMC-1), it is reasonable to extend such searches to other PAHs within the same source. In this work, we report a rotational spectroscopy study of commercially available fluoranthene (FA) and its synthesized cyano derivative, 3-cyanofluoranthene (3-CNFA), using chirped-pulse Fourier-transform microwave spectroscopy. The analysis of the rotational spectra, supported by quantum chemical calculations, yielded molecular parameters for the parent species of both FA and 3-CNFA molecules. The experimental data of 3-CNFA were later used for its astronomical search in TMC-1 with the QUIJOTE line survey but proved unsuccessful. Despite the nondetection of 3-CNFA in this source, the upper limit to its abundance was established. The experimental data will support future astronomical searches in the ISM.

## Graphical abstract

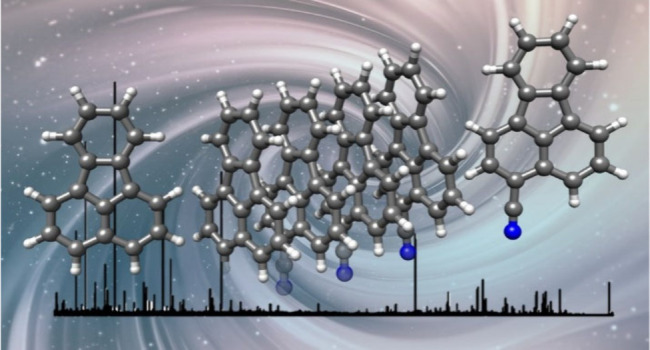

Polycyclic aromatic hydrocarbons (PAHs) are a class of widespread organic molecules found in chemical environments ranging from the Earth’s atmosphere to interstellar space.^1^ On Earth, they are carcinogenic byproducts of incomplete combustions, while in astronomical sources they are believed to be responsible for a set of prominent infrared emission bands observed within many cosmic objects. The presence of PAHs in both terrestrial and astrophysical contexts makes their chemistry relevant to environmental health and astrochemistry. The recent detection of specific PAHs in regions far beyond the Solar System has renewed interest in understanding their formation and destruction routes, along with their spectroscopic signatures.

The PAH hypothesis was first proposed in the mid-1980s, suggesting that the unidentified infrared emission features observed in the interstellar medium (ISM) arise from large PAH molecules.^2,3^ These compounds are estimated to contain about 20% of the elemental carbon in the ISM, representing the most abundant organic molecules in space. Although this hypothesis has gained broad acceptance, the lack of direct detections of individual PAHs has long been a major limitation. Most interstellar molecules, around 90% of those known, have been identified through their pure rotational spectrum via radio astronomy.^4^ Rotational detections of PAHs are challenging because their high molecular symmetry produces very small or zero permanent electric dipole moments, weakening their rotational transitions. The first confirmed detection of an individual PAH, indene (C_9_H_8_), in the cold pre-stellar core TMC-1 in 2021 represented a turning point.^5,6^ Recent detections of other PAHs and their cyano-substituted derivatives, enabled by the high sensitivity of the QUIJOTE (Q-band Ultrasensitive Inspection Journey to the Obscure TMC-1 Environment) and GOTHAM (GBT Observations of TMC-1: Hunting for Aromatic Molecules) radio telescope surveys, indicate that aromatic rings are abundant species in cold molecular clouds.^7^ In addition to indene, two other nonfunctionalized PAHs have been discovered in TMC-1, phenalene (C_13_H_10_)^8^ and 1*H*-cyclopent­[*cd*]­indene (C_11_H_8_).^9^ Some of the cyano derivatives of PAHs detected to date include indene,^10^ naphthalene^11^ and acenaphthylene.^12,13^ The cyano group increases the molecular dipole moment, making these derivatives easier to detect and valuable as observational tracers of their nonpolar parent PAHs. Most recently, larger species such as the four-ring isomers 1-, 2-, and 4-cyanopyrene (C_16_H_9_CN)^14,15^ and the seven-ring cyanocoronene^16^ PAHs were discovered in the GOTHAM observations. These discoveries demonstrate the growing effectiveness of laboratory rotational spectroscopy and coordinated astronomical observations in identifying new aromatic molecules in space.

Fluoranthene (C_16_H_10_, hereafter FA) is a four-ring PAH of particular interest as a potential interstellar molecule due to its structure combining arenes and a five-membered ring (Figure 1). FA is also a structural isomer of pyrene; nevertheless, in contrast to the higher symmetric structure of pyrene, it can be described as a fusion of a naphthalene and benzene moieties through a shared five-membered ring. This arrangement of fused rings gives the molecule a sizeable permanent dipole moment, enabling its pure rotational spectrum to be amenable via radio astronomy. Different studies suggest that neutral molecules of this size and photostability contribute significantly to the population of PAHs responsible for the aromatic infrared bands (AIBs) observed across the interstellar medium.^17^ Its formation could occur through gas-phase reactions involving smaller aromatic precursors (“bottom-up scenario”) or through the photochemical processing of larger carbonaceous species (“top-down scenario”) in the cold and shielded regions of dark clouds. Observational evidence from recent studies supports a “bottom-up” approach as being consistent with the PAH identifications and abundances in TMC-1.^5,18^ In this sense FA could represent a significant chemical milestone between the planar PAHs and the nonplanar geodesic polyarenes. Lately, several analytical techniques have detected PAHs in different types of the primitive carbonaceous chondrites (CCs) Murchison and Orgueil and in samples from comet 81P/Wild 2 collected by the *Stardust* mission.^19^ Among these, two C_16_H_10_ isomers, FA and pyrene, together with phenanthrene (C_14_H_10_), have been identified as the most abundant compounds.^19,20^ A recent detection in samples from the asteroid Ryugu also provides new insight into possible formation pathways in the ISM.^21,22^ Carbon-13 isotopic analysis of Ryugu PAHs^21^ shows that three-ring species such as anthracene and phenanthrene were formed at high temperatures (>1,000 K). In contrast, two- and four-ring PAHs such as naphthalene, FA, and pyrene (the most abundant PAH on Ryugu), were formed at low temperatures (∼10 K), in agreement with the detection of two- and four-ring PAHs in the cold TMC-1 by radio observations. The highest Δ_2_ × ^13^C (doubly-^13^C substituted compositions) value for the Murchison FA matches the acetylene equilibrium value at ∼10 K, suggesting that most of the FA was synthesized in the ISM. Consequently, FA represents a candidate for both the formation and survival of PAHs in space.

**Figure 1:**
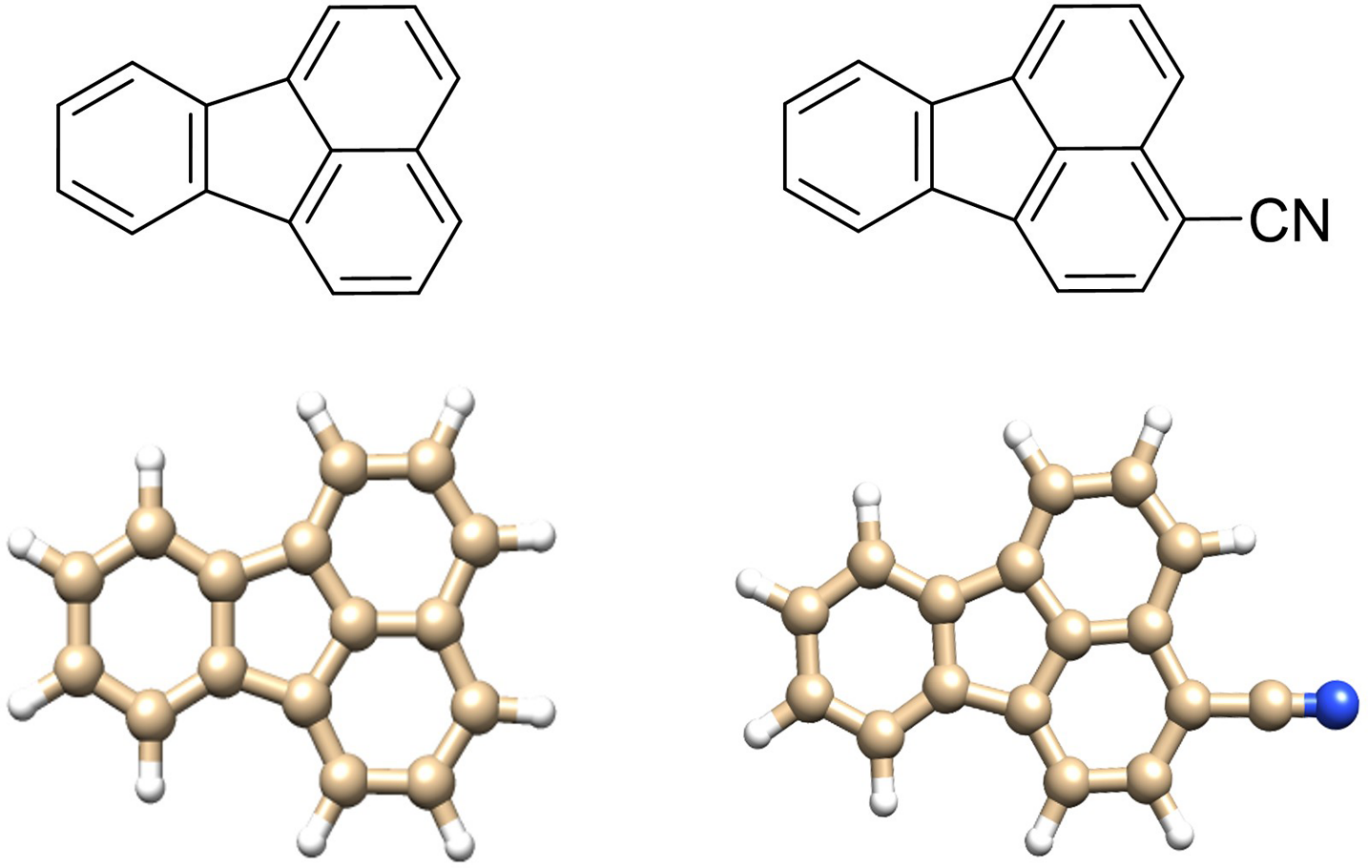
Schematic (top) and molecular (bottom) structures at the B3LYP/6-311++G­(d,p) level of theory of FA (left) and 3-CNFA (right).

The introduction of functional groups, such as the cyano substituent in FA, further enhances the dipole moment and the detectability of related species, providing a pathway for observational confirmation. Recently, Wenzel et al.^15^ suggested that cyanopyrene isomers form via direct addition of the cyano group to pyrene. Detection of at least one isomer of cyanofluoranthene (hereafter CNFA, see Figure 1 and Figure S1 of Supporting Information) would therefore not only confirm the presence of the FA skeleton in space but also corroborate active nitrile chemistry and the incorporation of nitrogen into aromatic networks. In addition, detecting both FA and CNFA would provide a means to quantify the fraction of pure PAHs converted into their cyano derivatives and to estimate parent PAH abundances even when the parent species itself is not directly detectable.^10^ Considering this, CNFA also represents an important target aimed at expanding the molecular inventory of aromatic species in space and understanding the chemical evolution of complex organics in the ISM. To facilitate interstellar searches for FA and CNFA, their pure rotational spectra must first be investigated in the laboratory. While FA has already been studied using structural techniques (including vibrational^23^ and electronic^24^ spectroscopies and X-ray^25^ diffraction), to the best of our knowledge, no rotational spectroscopic data have been reported in the literature for either FA or CNFA; in fact, no isomer of CNFA is commercially available. Herein, we present a high-resolution rotational study of FA and 3-CNFA using broadband chirped-pulse Fourier transform microwave (CP-FTMW) spectroscopy. Analysis of the experimental spectra of FA and 3-CNFA yielded precise spectroscopic constants, enabling accurate frequency predictions for astronomical searches.

A commercially available sample of FA was purchased (>98% purity) while 3-CNFA was synthesized for this work as detailed in Experimental Methods and the Supporting Information. Since FA and 3-CNFA are solids at room temperature, experiments on molecular jet expansions were conducted using a heating nozzle with Ne as carrier gas (see Experimental Methods).

The analysis of the rotational spectra of FA and 3-CNFA was assisted by quantum chemical calculations using density functional theory and Møller–Plesset MP2 computations (see Table 1, Table S1 and Experimental Methods). The theoretical values of the rotational constants in Table 1 indicate that both FA and 3-CNFA are asymmetric rotors with Ray’s asymmetry parameters (*κ*) of −0.55 and −0.80, respectively, placing 3-CNFA closer to the prolate limit. Both species exhibit *μ*_*a*_-type spectra, with dipole moment components of 0.4 D for FA and 5.1 D for 3-CNFA, respectively. 3-CNFA could also have a *μ*_*b*_-type spectrum as the value of this dipole moment component is approximately 0.2 D. The broadband spectrum of FA, recorded in the 2–8 GHz frequency region, is shown in Figure 2 and has a line intensity profile corresponding to a rotational temperature of ca. 2 K. Attempts to measure additional rotational transitions above 8 GHz were unsuccessful, as no lines were observed owing to the low dipole moment of FA and the reduced population of higher rotational states at that temperature. A total of 65 *μ*_*a*_-type R-branch rotational transitions with quantum numbers 2 ≤ *J* ≤ 11 and 0 ≤ *K*_*a*_ ≤ 7 could be measured and fit to experimental accuracy (10 kHz) using a rigid rotor Hamiltonian with Pickett’s CALPGM programs.^26^ As can be observed, the molecule is quite rigid and no centrifugal distortion constants could be determined for the present set of transitions. A list of the measured frequencies and the derived rotational constants (*A*, *B* and *C*) are collected in the Supporting Information and Table 1, respectively. The broadband spectrum of 3-CNFA was recorded in the 2–10 GHz frequency region, and a section is shown in Figure S2 of the Supporting Information. The presence of a ^14^N-nitrogen nucleus in this molecule, which has a nonzero nuclear spin (*I* = 1), leads to the splitting of each rotational transition into several hyperfine components due to nuclear quadrupole coupling.^27^ However, as illustrated in Figure 3, the quadrupole hyperfine structure collapses at higher frequencies, preventing the resolution of individual hyperfine components and leading to an increase in the intensity of these transitions. These frequencies and those corresponding to the resolved hyperfine components were globally fit to an experimental accuracy of 10 kHz using Watson’s *A*-reduced semirigid Hamiltonian (*III* ^*l*^ representation).^28^ Finally, a total of 170 lines belonging to *μ*_*a*_-type R-branch rotational transitions with quantum numbers 3 ≤ *J* ≤ 21 and 0 ≤ *K*_*a*_ ≤ 6 could be measured; however, no *b*-type transitions were detected due to the small value of the predicted dipole moment component. The list of lines observed for 3-CNFA is given in the Supporting Information. The derived experimental values of 3-CNFA are shown in Table 1, and they include the rotational constants, two centrifugal distortion constants, and two elements of the ^14^N nuclear quadrupole coupling tensor. As shown in Table S1 of the Supporting Information, the rotational and nuclear quadrupole coupling constants only match with isomer 3-CNFA. The rigidity of these molecules produces a very good agreement (relative differences <0.2% for FA and <0.1% for 3-CNFA) between the ground-state rotational constants and those predicted from quantum chemical calculations at B3LYP/6-311++G­(d,p) level of theory. Larger discrepancies (<0.6% for FA and <0.7% for 3-CNFA) were observed when compared to rotational constants predicted using the MP2 method.

**Table 1:** Rotational Parameters for the FA and 3-CNFA Molecules^a^

	**FA**		**3-CNFA**	
**Parameter**	**Theory**	**Experiment**	**Theory**	**Experiment**
*A*/MHz^b^	1036.6	1034.7575(16)^g^	989.3	989.6500(49)
*B*/MHz	492.2	492.916010(93)	312.9	313.33953(29)
*C*/MHz	333.7	333.969937(75)	237.7	238.04796(22)
*Δ_J_*/kHz	0.0087		0.0115	0.01030(57)
*Δ_JK_*/kHz	–0.0148		–0.0263	–0.02444(83)
*Δ_K_*/kHz	0.0069		0.0152	[0.0152]^h^
*δ_J_*/kHz	–0.0026		–0.00516	[−0.00516]
*δ_K_*/kHz	0.0026		0.00794	[0.00794]
χ_*aa*_/MHz			–4.4	–4.197(38)
χ_*bb*_/MHz			2.5	2.07(11)
|*μ_a_* |, |*μ_b_* |, |*μ_c_* |/D	0.4, 0.0, 0.0	y, n, n	5.1, 0.2, 0.0	y, n, n
*N^c^*		65		170
σ^d^/KHz		3.5		10.5
κ^e^	–0.55	-0.55	-0.80	-0.80
*Δ_c_* ^f^/u Å^2^	0.0	–0.4405(20)	0.0	–0.5308(60)

a The experimental values correspond to the vibrational ground-state while the theoretical values correspond to the equilibrium configuration calculated at the B3LYP/6-311++G­(d,p) level of theory.

b *A*, *B*, and *C* are the rotational constants; *Δ* *_J_*, *Δ_JK_*, *Δ_K_, *δ_J_** and *δ_K_* are the Watson’s A-reduction quartic centrifugal distortion constants; χ_aa_ and χ_bb_ are elements of the nuclear quadrupole tensor and |*μ*_*a*_|, |*μ*_*b*_|, and |*μ*_*c*_| are the absolute values of the electric dipole moment components along the principal inertial axes; y and n (yes and no), indicate whether a-, b- or c-type transitions are observed or not.

c Number of fitted transitions.

d rms of the fit.

e Ray’s parameter defined as (2*B* – *A* – *C*)/(*A* – *C*).

f Inertia defect, Δ*c* = *Ic* – *Ib* – *Ia*. The conversion factor is 505379.1 MHz u Å^2^.

g Standard error in parentheses in units of the last digit.

h Parameters in square brackets were kept fixed in the fit.

**Figure 2:**
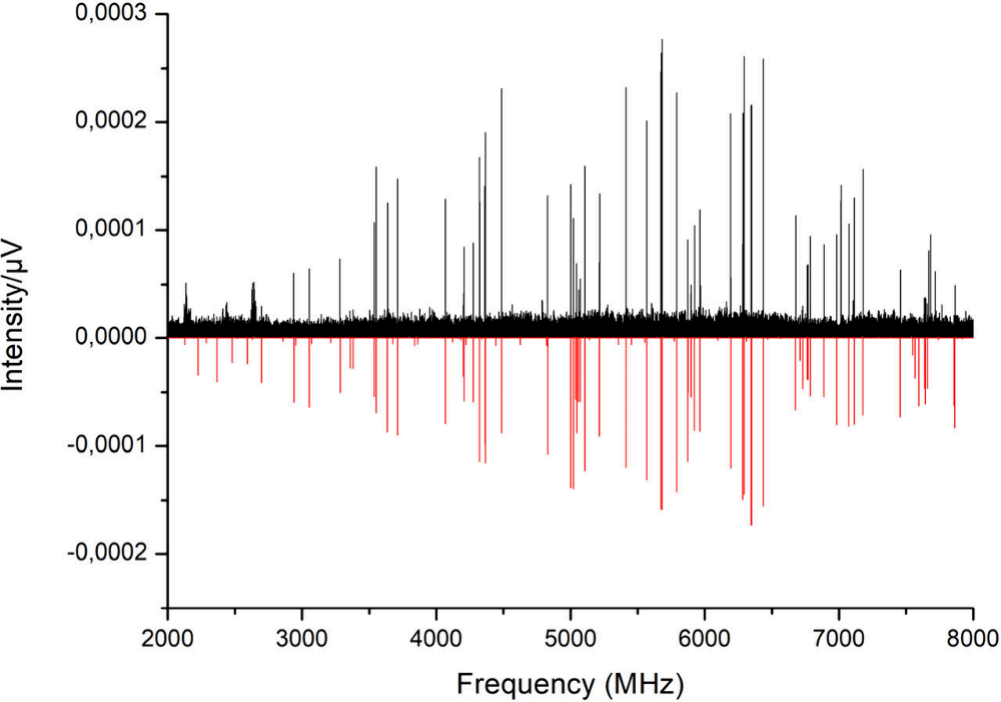
Broadband microwave spectrum of FA in the frequency region 2–8 GHz, represented by the positive black trace. The negative red trace is a simulation at 2 K using the fitted rotational parameters of Table 1.

**Figure 3:**
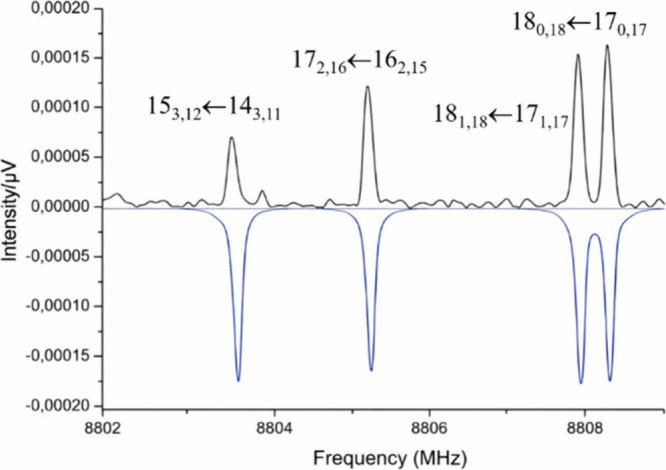
A section of the broadband microwave spectrum of 3-CNFA showing the collapsed nuclear quadrupole hyperfine structure mentioned in the text. The negative blue trace is a simulation at 2 K using the fitted rotational parameters of Table 1. A Gaussian line profile and a typical full width at half-maximum (FWHM) line width of ca. 100 kHz were used for the simulation.

Initial information on the molecular structure could be derived from the experimental inertial defects (Table 1), which are calculated from the determined principal moments of inertia according to *Δ*_*c*_ = *I*_*c*_ – *I*_*b*_ – *I*_*a*_, where *I*_*α*_ (*α* = *a*, *b*, *c*) are the principal moments of inertia of the molecule (Figure S3 of Supporting Information). The inertial defect is zero for a rigid planar molecule in its equilibrium structure, while for a nonplanar molecule it is negative.^27^ For the molecules reported here, we would expect an inertial defect of zero; however, we have determined the inertial defects to be −0.4405(20) and −0.5308(60) u Å^2^, for FA and 3-CNFA, respectively. These deviations from zero can be attributed to zero-point molecular vibrations. Indeed, in-plane vibrations make a small, positive contribution to the inertial defect, while out-of-plane (OOP) vibrational modes make a small negative contribution.^29^ This is in accordance with the values of the inertial defect determined for other PAHs such as 1-fluoronaphthalene (−0.144 u Å^2^),^30^ azulene (−0.151 u Å^2^),^31^ acenaphthylene (−0.192 u Å^2^),^31^ and pyrene (−0.646 u Å^2^).^32^ Considering the larger value of the inertial defect of the planar molecule pyrene, we can conclude that FA and 3-CNFA are also planar. To confirm this, anharmonic vibrational modes were calculated at the B3LYP/6-311++G­(d,p) level of theory. The results show that the three lowest vibrational modes of FA, predicted at 100, 120, and 163 cm^–1^, correspond to OOP motions. Similar results were obtained for 3-CNFA: the two lowest vibrational modes, at 62 and 116 cm^–1^, are also OOP motions, whereas the mode at 121 cm^–1^ corresponds to the first in-plane vibration, with the main contribution arising from the nitrile group.

The molecular constants derived in this work (Table 1) for FA and 3-CNFA molecules serve as a good guide for their astronomical searches. The frequency predictions for both species were obtained and implemented in the MADEX code.^33^ These predictions in the K band region (18 to 26 GHz) have accuracies around 40–60 kHz for the *a*-type transitions with *K*_*a*_ = 0, 1 and 2. However, this worsens in the Q band region (30–50 GHz), where the accuracies are larger than 200 kHz. This situation and the fact that the intensity of the FA and 3-CNFA lines is predicted to be larger at lower frequencies led us to perform the astronomical search for these species in our K-band survey. The astronomical observations presented in this work are from the ongoing K-band line survey of TMC-1 ^13,34^ (α_2000_ = 4^h^ 41^m^ 41.9^s^; δ_2000_ = 25° 41′ 27″) obtained with a new receiver built for the Yebes 40m radio telescope^35^ and optimized in the 18–32 GHz frequency range. The radio telescope observations were taken with different local oscillator frequencies to avoid ghost signals that might be produced in the down-conversion chain. The adopted velocity with respect to the Local Standard of Rest (v_LSR_) is 5.83 km s^–1^.^36^ The observing procedure and the data analysis are similar to those used in the Q band,^34,37^ that is, frequency switching with two different throws. A total of 226 hours of on-source observing time was accomplished in the 18–26 GHz range producing a sensitivity between 0.2–0.4 mK.

For the astronomical search of 3-CNFA in TMC-1 we selected a total of nineteen transitions with *J* values between 35 and 45 and *K*_*a*_ values ranging from 0 to 4. These lines are the most promising candidates based on the predicted intensities and the sensitivity of the different frequency regions of our survey. All expected lines should be in emission. As can be seen in the Figure 4, six lines have a counterpart in the data (green stars). However, there are nine lines that are not detected (red stars) while four lines are blended with transitions of other known species (blue stars). Considering these observational facts, we can conclude that the 3-CNFA molecule is not detected at the present level of sensitivity of our astronomical data. We have assumed the physical parameters that we adopted in our previous works of TMC-1 ^13,18^ (see also Appendix C of ref 38), i.e., *T*_rot_ = 8 K, a diameter for the source of 70″, and a line width of 0.5 km/s. We found that the column density that best fits the selected lines is 1.5 × 10^12^ cm^–2^. The predicted line intensities from the model, together with the data sensitivity, should be enough to have a positive detection of all selected lines. Hence, we must conclude that too many transitions are missing to claim any reasonable identification. As commented above, the frequency accuracy of the predictions, which are based on the laboratory molecular constants and their covariance matrix, is only 40 kHz for the rotational transitions within the K band. We have explored the possibility that the missing lines could be within 100 kHz of the predicted frequencies. However, this still did not yield a good match. It is worth noting that by doing a blind stacking of the data, i.e., without careful elimination of lines blended with other features, we could get a positive detection. However, it is difficult to believe such a detection as severe blending does occur for some lines (see Figure 4). When the lines affected by blending are removed, the stacking detection is much less credible. From the stacked data of unblended lines, we could provide a 3σ upper limit to the column density of 3-CNFA of 10^12^ cm^–2^. Despite having predictions for the frequencies of rotational transitions for FA that make it possible to search for it in TMC-1, its dipole moment is very low and, therefore, the derived upper limit to its column density does not provide any significant constraint to the chemistry of PAHs in TMC-1. The derived upper limit to the column density of 3-CNFA are of the order of the values derived for the four isomers of cyanoacenaphthylene recently detected in TMC-1.^12,13^ However, the abundance of 3-CNFA is at least a factor 10 below that of indene^5^ or that of phenalene.^8^ Hence, the detection of these PAHs in a cold prestellar core indicates that a bottom-up process, still to be identified, is probably at work in these cold and low-density objects.

**Figure 4:**
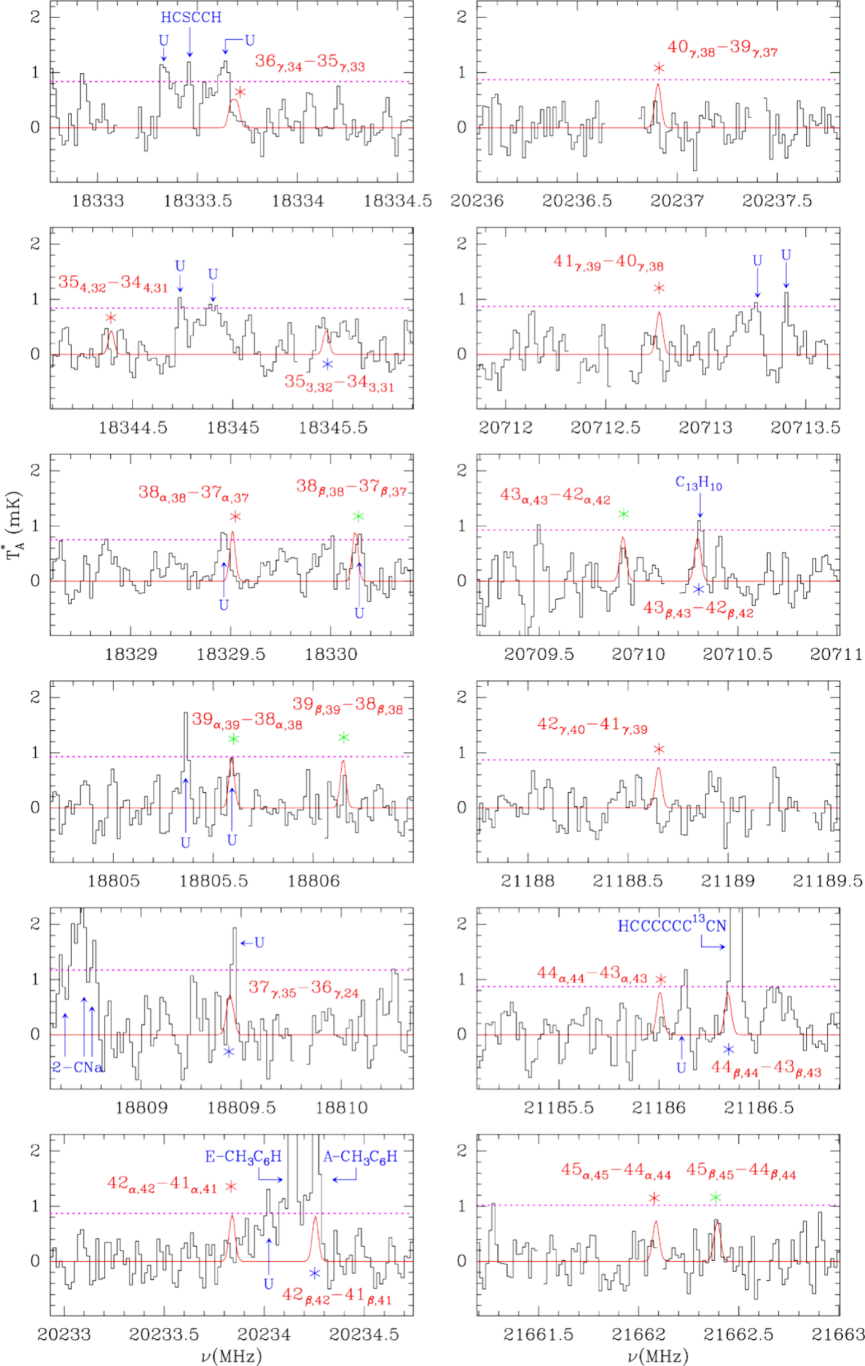
Spectra of TMC-1 in the K band from the QUIJOTE survey at the frequencies of some of the most intense predicted lines of 3-CNFA. Quantum numbers for each rotational transition are indicated in each panel. The labels α, β, and γ correspond to unresolved doublets with *K*_*a*_ = 0, 1, *K*_*a*_ = 1, 2, and *K*_*a*_ = 2, 3, respectively. We show as a red trace the synthetic line profiles calculated for the column density upper limit given in the text. As observed, 3-CNFA is not detected in TMC-1 at the current level of sensitivity of our data (the horizontal dashed purple line represents the 3σ level). Red and green stars indicate transitions without or with a possible counterpart in the data. Blue stars correspond to transitions blended with lines from other species. Lines above 3σ that arise from other species are indicated. U labels correspond to unknown features.

In summary, this study presents synthetic, spectroscopic, and astronomical investigations of FA and 3-CNFA. The spectroscopic measurements yield the first high-resolution gas-phase structural information for these molecules. Rotational constants, centrifugal distortion constants, and nuclear quadrupole coupling constants were derived, enabling a radio astronomical search of these molecules in the K band. Although no detection could be made in this band, upper limits to their abundances in this source were established. This study highlights the increasing capability of chirped-pulse broadband microwave spectroscopy, combined with targeted astronomical observations, to support ongoing and future astronomical searches for aromatic molecules in space.

## Experimental Methods

### Sample Synthesis

The synthesis of 3-CNFA was achieved in two steps starting from commercially available FA (see Supporting Information). It started with the treatment of FA with *N*-bromosuccinimide (NBS) allowing for the quantitative isolation of 3-bromofluoranthene, which upon cyanodebromination with CuCN produced 3-cyanofluoranthene in good yield.

### Quantum Chemical Calculations

To support spectral assignment, quantum chemical calculations were performed using the Gaussian16 program package^39^ to obtain the FA and 3-CNFA optimized structures. The B3LYP hybrid density functional^40^ together with the Pople triple-ζ basis set 6-311++G­(d,p)^41^ was used to derive the rotational constants, electric dipole moment components and nuclear quadrupole coupling constants for FA and the isomers of CNFA (Table 1 and Table S1 of the Supporting Information). The Moller–Plesset (MP2) perturbation theory^42^ in the frozen core approximation method was checked for consistency (Table S1 of the Supporting Information), using the same basis set. Anharmonic vibrational frequencies were computed at the B3LYP level of theory to estimate the centrifugal distortion constants and rovibrational contributions for FA and 3-CNFA.

### CP-FTMW Spectroscopy

FA and 3-CNFA species were studied in a broadband chirped-pulsed Fourier-transform microwave (CP-FTMW) spectrometer at the Universidad de Valladolid operating from 2 to 18 GHz.^43,44^ The solid samples of FA and 3-CNFA were vaporized at 160 and 135 °C, respectively, in a solenoid-driven pulsed injector and seeded with an inert carrier gas (neon, 3 bar) to generate molecular jet pulses (typ. 900 *μ*s) with effective rotational temperatures of 2 K. The molecules of each gas pulse were polarized using a series of 20 microwave pulses with a duration of 4 *μ*s. The measurement was performed at a repetition rate of 5 Hz, giving an effective overall repetition rate of 100 Hz. The chirp pulses were generated with an arbitrary waveform generator (Tektronix AWG 70002A, 25 GS/s), amplified by a solid-state amplifier (Qorvo QPB0220N, with an output power of 54 dBm) and broadcasted through a horn antenna perpendicular to the propagation of the jet expansion. A molecular transient emission, spanning 20 *μ*s, was then detected through a second receiving horn and amplified by a low-noise MW amplifier (Miteq LNAS-55-01001800-22-10P). A total of 2000 k and 3200 k FIDs per measurement for FA and 3-CNFA, respectively, were finally co-added on a digital oscilloscope (Tektronix DPO 73304DX, 100 GS/s) and Fourier transformed to finally obtain the resonance frequencies of the rotational transitions. Application of a Kaiser–Bessel apodization window yielded a typical full width at half-maximum (FWHM) line width of ca. 100 kHz. The accuracy of the frequency measurements is better than 10 kHz. All frequency components were referenced to a Rb standard.

### Radio Astronomical Observations

The methodology and observing procedures used to get the QUIJOTE data have been described by Cernicharo et al.^5,13,37^ The QUIJOTE data correspond to a total of 1509 hours of observing time in the source.

## Supplementary Material





## Notes

Molecular structures and theoretical spectroscopic parameters of all the isomers of CNFA; broadband microwave spectrum; list of the measured rotational transitions for FA and 3-CNFA; Cartesian coordinates from the optimized structures FA and 3-CNFA; text including synthesis and characterization data; NMR spectra (PDF)
Transparent Peer Review report available (PDF)
