# Supplementary material for: Rotational Characterization of Four-Ring Polycyclic Aromatic Hydrocarbons: Toward the Detection of Fluoranthene and Cyanofluoranthene in Space
Source: J Phys Chem Lett. 2026 Apr 17;17(17):5012–20. doi: 10.1021/acs.jpclett.6c00413 (PMC13137236; doi:10.1021/acs.jpclett.6c00413)
Supplement: Supplementary file 1 [file jz6c00413_si_001.pdf]

Rotational Characterization of Four-ring Polycyclic Aromatic Hydrocarbons: Towards the detection of Fluoranthene and Cyanofluoranthene in Space

Supporting Information

Daniel Villar-Castro,[a] Carlos Cabezas,[b] Amanda L. Steber,[c] José R. Morán,[c] Selene de la Fuente,[c] Farha. S. Hussain,[c] Dolores Pérez,[a] Alberto Lesarri,[c] José Cernicharo,[b] Cristóbal Pérez,[a,c] Isabel Peña[,c]*

[a] Centro Singular de Investigación en Química Biolóxica e Materiais Moleculares (CiQUS) and Departamento de Química Orgánica, Universidade de Santiago de Compostela, 15782 Santiago de Compostela, Spain

[b] Instituto de Física Fundamental, CSIC, C/ Serrano 123, 28006 Madrid, Spain

[c] Departamento de Química Física y Química Inorgánica, Facultad de Ciencias. - I.U. CINQUIMA, Universidad de Valladolid, 47011 Valladolid, Spain

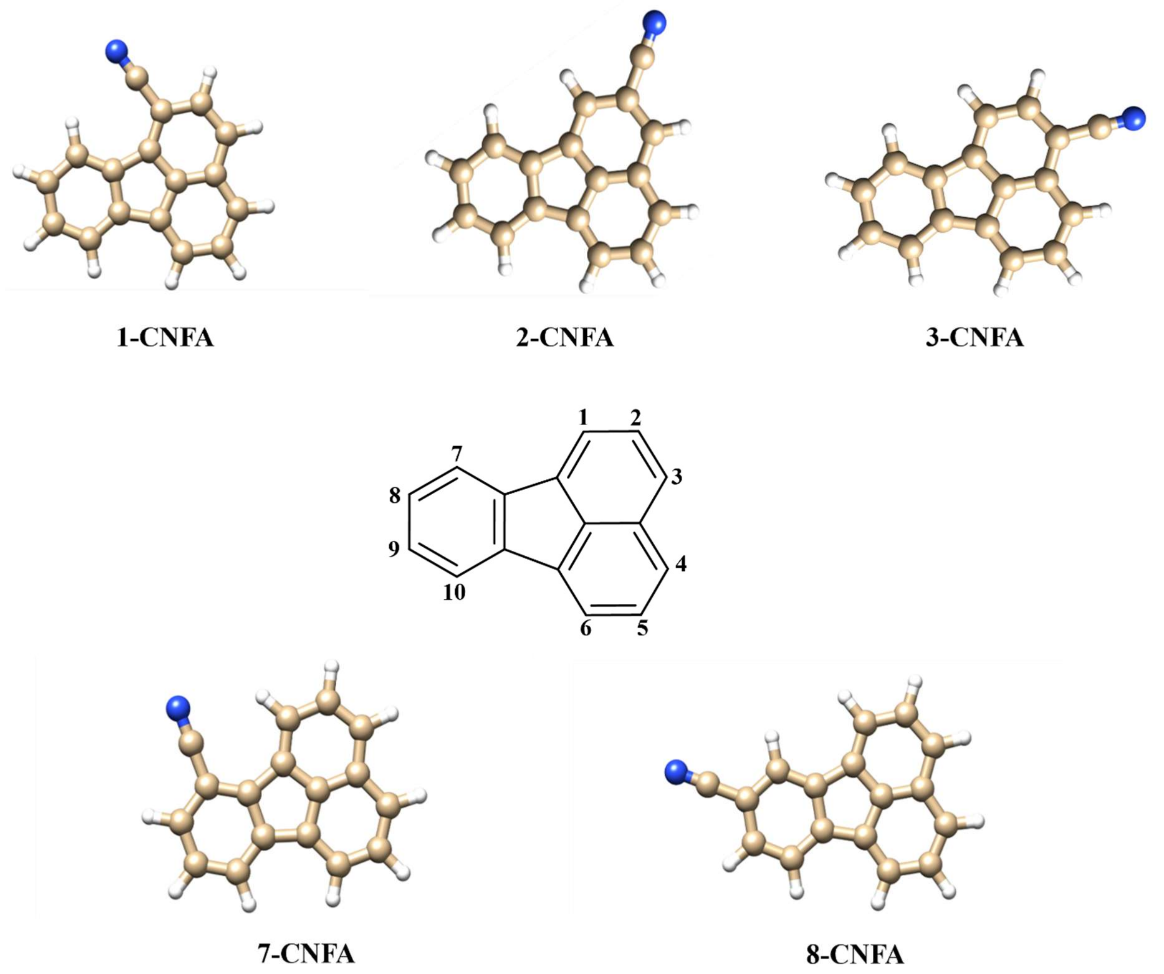

Figure S1. Schematic structure of FA (central) and its cyano derivatives (CNFA) optimized at the B3LYP-6-311++G(d,p) level of theory. The atom numbering of FA indicates the possible nitrile substitution sites corresponding to the five CNFA isomers. The following positions are symmetry-equivalent: 1–6, 2–5, 3–4, 7–10, and 8–9.

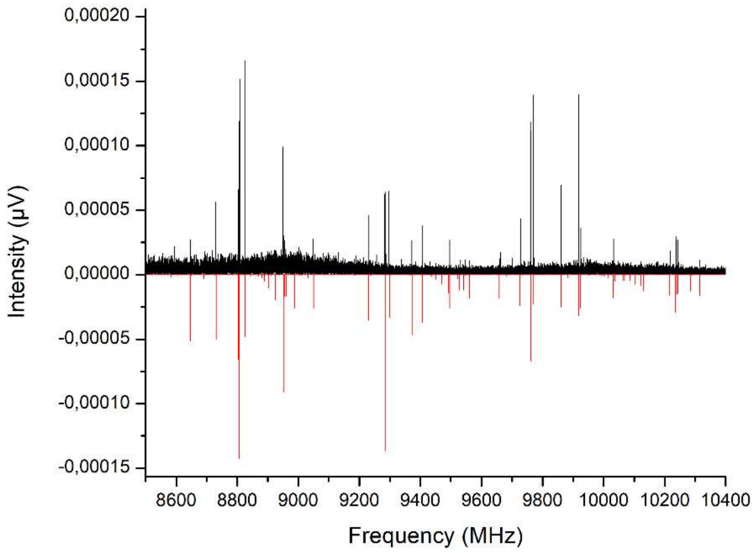

Figure S2. A section of the broadband microwave spectrum of 3-CNFA recorded in the frequency region 2-10 GHz (black trace). The negative red trace is a simulation at 2 K using the fitted rotational parameters of Table 1.

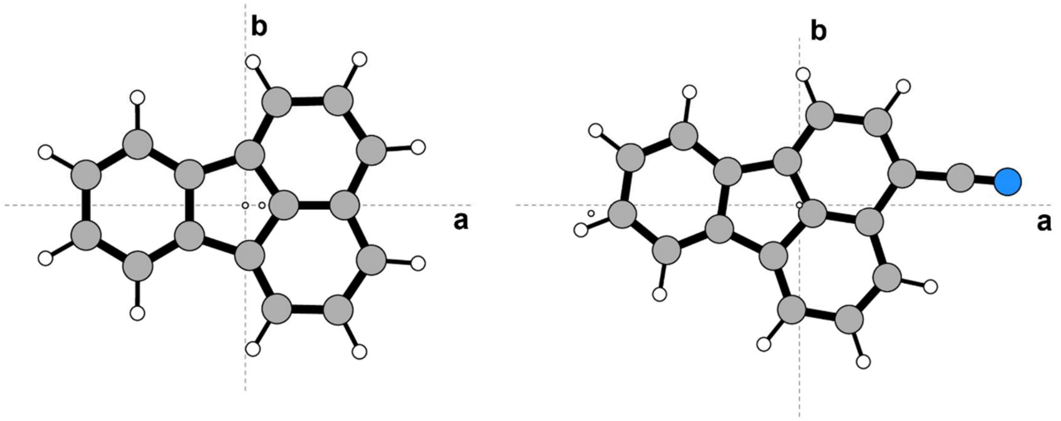

Figure S3. Molecular structures of FA and 3-CNFA showing the principal inertial axes *a* and *b*.

Table S1. Calculated spectroscopic parameters for the FA and the isomers of CNFA at the MP2/6-311++G(d,p) and B3LYP/6-311++G(d,p) levels of theory.

	FA		1-CNFA		2-CNFA		3-CNFA		7-CNFA		8-CNFA	
**Parameters**	**MP2**	**B3LYP**	**MP2**	**B3LYP**	**MP2**	**B3LYP**	**MP2**	**B3LYP**	**MP2**	**B3LYP**	**MP2**	**B3LYP**
A/ MHz[a]	1029.4	1036.6	575.3	574.6	687.7	693.1	985.9	989.4	726.9	735.5	968.5	975.6
B/ MHz	490.2	492.2	482.9	486.4	354.9	356.5	311.1	312.9	404.1	402.7	287.8	289.3
C/ MHz	332.1	333.7	262.5	263.4	234.1	235.4	236.5	237.7	259.7	260.2	221.9	223.3
χ_{aa}/ MHz	-	-	-0.9	-0.6	-2.0	-2.4	-3.6	-4.4	1.1	1.4	-3.0	-3.7
χ_{bb}/ MHz	-	-	-0.9	-1.4	0.2	0.4	1.8	2.5	-2.9	-3.3	1.2	1.7
$\|\mu_a\|$/ D	0.4	0.4	2.9	2.6	4.2	4.5	4.5	5.1	2.8	3.1	5.5	4.5
$\|\mu_b\|$/ D	0.0	0.0	3.6	4.1	2.8	3.0	0.2	0.2	3.4	3.5	1.2	3.0
$\|\mu_c\|$/ D	0.0	0.0	0.0	0.0	0.0	0.0	0.0	0.0	0.0	0.0	0.0	0.0

[a] A, B, C are the rotational constants; χ_{aa} and χ_{bb} are the nuclear quadrupole constants; $|\mu_a|$, $|\mu_b|$ and $|\mu_c|$ are the absolute values of the electric dipole moment components along the principal inertial axes.

Table S2. Measured frequencies and residuals (in MHz) of the rotational transitions of the FA species using the CP-FTMW spectrometer in Valladolid.

J'	K_a'	K_c'	J''	K_a''	K_c''	ν_{obs}	$\nu_{obs}-\nu_{calc}$
3	1	3	2	1	2	2224.8951	-0.0069
3	0	3	2	0	2	2366.6613	-0.0034
3	2	2	2	2	1	2480.6544	-0.0034
3	2	1	2	2	0	2594.6538	0.0029
3	1	2	2	1	1	2698.0903	-0.0018
4	1	4	3	1	3	2938.7758	-0.0014
4	0	4	3	0	3	3054.8491	-0.0003
4	2	3	3	2	2	3283.9019	0.0027
4	3	2	3	3	1	3358.4252	0.0072
4	2	2	3	2	1	3536.2329	0.0044
4	1	3	3	1	2	3553.2196	0.0071
5	1	5	4	1	4	3637.2025	0.0002
5	0	5	4	0	4	3712.6300	-0.0028
5	2	4	4	2	3	4067.6775	-0.0032
5	4	2	4	4	1	4201.1790	0.0003
5	3	3	4	3	2	4205.5883	-0.0060
5	3	2	4	3	1	4276.3219	0.0063
6	1	6	5	1	5	4323.3557	-0.0008
5	1	4	4	1	3	4361.1820	0.0035
6	0	6	5	0	5	4365.0554	0.0009
5	2	3	4	2	2	4486.6095	0.0068
6	2	5	5	2	4	4829.0159	-0.0018
7	1	7	6	1	6	5001.1724	-0.0005
7	0	7	6	0	6	5021.9633	-0.0012
6	3	4	5	3	3	5045.9673	-0.0059
6	4	3	5	4	2	5059.9267	0.0001
6	4	2	5	4	1	5072.4899	-0.0011
6	1	5	5	1	4	5106.9574	-0.0007
6	3	3	5	3	2	5215.7250	0.0028
6	2	4	5	2	3	5412.7103	-0.0014
7	2	6	6	2	5	5567.0703	0.0039
8	1	8	7	1	7	5674.0192	-0.0018
8	0	8	7	0	7	5683.7351	0.0056
7	1	6	6	1	5	5791.1927	-0.0026
7	3	5	6	3	4	5872.0677	0.0091

J'	K_a'	K_c'	J''	K_a''	K_c''	ν_{obs}	$\nu_{obs}-\nu_{calc}$
7	6	2	6	6	1	5873.9698	-0.0025
7	4	4	6	4	3	5923.5626	-0.0036
7	4	3	6	4	2	5963.3862	-0.0004
7	3	4	6	3	3	6192.8971	-0.0042
8	2	7	7	2	6	6283.5220	-0.0010
7	2	5	6	2	4	6293.4476	-0.0024
9	1	9	8	1	8	6344.2098	-0.0001
9	0	9	8	0	8	6348.5495	0.0020
8	1	7	7	1	6	6437.2345	0.0023
8	3	6	7	3	5	6677.4760	-0.0014
8	7	2	7	7	1	6710.3814	-0.0061
8	5	4	7	5	3	6762.6770	-0.0033
8	5	3	7	5	2	6769.4961	0.0027
8	4	5	7	4	4	6787.3077	0.0037
8	4	4	7	4	3	6888.0272	0.0013
9	2	8	8	2	7	6982.2051	-0.0011
10	1	10	9	1	9	7013.0940	0.0001
10	0	10	9	0	9	7014.9705	-0.0012
9	1	8	8	1	7	7074.2171	-0.0018
8	2	6	7	2	5	7113.9507	0.0042
8	3	5	7	3	4	7179.0143	-0.0045
9	3	7	8	3	6	7458.0470	0.0001
9	7	2	8	7	1	7565.5773	-0.0007
9	5	5	8	5	4	7636.0160	-0.0035
9	4	6	8	4	5	7644.2705	0.0054
10	2	9	9	2	8	7667.9167	-0.0000
11	1	11	10	1	10	7681.3834	0.0001
11	0	11	10	0	10	7682.1790	0.0019
10	1	9	9	1	8	7717.7960	0.0012
9	2	7	8	2	6	7863.1408	-0.0030

Table S3. Measured frequencies and residuals (in MHz) of the rotational transitions of the 3-CNFA species using the CP-FTMW spectrometer in Valladolid.

J'	K_a'	K_c'	F'	J''	K_a''	K_c''	F''	ν_{obs}	ν_{obs}-ν_{calc}
4	1	4	3	3	1	3	2	2043.9603	0.0408
4	1	4	5	3	1	3	4	2043.9603	-0.0146
4	0	4	-	3	0	3	-	2148.2476	0.0190
4	2	3	4	3	2	2	3	2200.5082	0.0055
4	2	3	5	3	2	2	4	2201.0755	0.0018
4	2	3	3	3	2	2	2	2201.2160	-0.0039
4	2	2	4	3	2	1	3	2257.8012	-0.0038
4	2	2	5	3	2	1	4	2258.3988	-0. 0141
4	2	2	3	3	2	1	2	2258.5613	0.0269
4	1	3	4	3	1	2	3	2343.2109	-0.0116
4	1	3	3	3	1	2	2	2343.3729	0.0424
4	1	3	5	3	1	2	4	2343.3729	-0.0133
5	1	5	6	4	1	4	5	2545.6550	0.0095
5	0	5	-	4	0	4	-	2647.8439	0.0070
5	3	3	5	4	3	2	4	2773.9889	0.0118
5	3	3	6	4	3	2	5	2774.6290	0.0040
5	3	3	4	4	3	2	3	2774.7588	-0.0245
5	3	2	5	4	3	1	4	2780.0642	-0.0127
5	3	2	6	4	3	1	5	2780.7370	0.0101
5	3	2	4	4	3	1	3	2780.9024	0.0169
5	2	3	5	4	2	2	4	2852.5206	-0.0075
5	2	3	4	4	2	2	3	2852.8122	-0.0545
5	1	4	5	4	1	3	4	2915.9109	-0.0080
5	1	4	4	4	1	3	3	2916.0081	0.0500
5	1	4	6	4	1	3	5	2916.0081	0.0026
6	1	6	-	5	1	5	-	3042.4794	-0.0062
6	0	6	-	5	0	5	-	3131.7344	0.0161
6	2	5	6	5	2	4	5	3281.5872	0.0048
6	2	5	7	5	2	4	6	3281.7581	0.0011
6	2	5	5	5	2	4	4	3281.7581	0.0024
6	3	4	6	5	3	3	5	3333.1331	0.0141
6	3	4	7	5	3	3	6	3333.5231	0.0236
6	3	4	5	5	3	3	4	3333.5231	-0.0351
6	3	3	6	5	3	2	5	3349.1363	0.0091
6	3	3	7	5	3	2	6	3349.5231	0.0116

J'	K_a'	K_c'	F'	J''	K_a''	K_c''	F''	ν_{obs}	ν_{obs}-ν_{calc}
6	3	3	5	5	3	2	4	3349.5231	-0.0477
6	2	4	6	5	2	3	5	3457.7419	-0.0094
6	2	4	5	5	2	3	4	3457.9313	-0.0030
6	2	4	7	5	2	3	6	3457.9313	-0.0037
6	1	5	-	5	1	4	-	3478.2113	-0.0134
7	1	7	-	6	1	6	-	3534.6977	-0.0238
7	0	7	-	6	0	6	-	3605.3082	0.0233
7	2	6	7	6	2	5	6	3813.8956	-0.0148
7	2	6	6	6	2	5	5	3814.0261	0.0142
7	2	6	8	6	2	5	7	3814.0261	0.0044
7	4	4	7	6	4	3	6	3888.4558	0.0028
7	4	4	8	6	4	3	7	3888.9020	0.0256
7	4	4	6	6	4	3	5	3888.9020	-0.0403
7	4	3	7	6	4	2	6	3889.8847	0.0043
7	4	3	8	6	4	2	7	3890.3215	0.0174
7	4	3	6	6	4	2	5	3890.3215	-0.0487
7	3	5	7	6	3	4	6	3892.3353	0.0023
7	3	5	8	6	3	4	7	3892.5891	0.0132
7	3	5	6	6	3	4	5	3892.5891	-0.0085
7	3	4	7	6	3	3	6	3927.3649	-0.0182
7	3	4	8	6	3	3	7	3927.6206	-0.0112
7	3	4	6	6	3	3	5	3927.6206	-0.0336
8	1	8	-	7	1	7	-	4022.8138	-0.0016
7	1	6	-	6	1	5	-	4026.6773	-0.0124
7	2	5	7	6	2	4	6	4066.9076	0.0009
7	2	5	6	6	2	4	5	4067.0094	-0.0042
7	2	5	8	6	2	4	7	4067.0094	-0.0143
8	0	8	-	7	0	7	-	4074.3539	0.0134
8	2	7	-	7	2	6	-	4339.9074	-0.0164
8	3	6	8	7	3	5	7	4450.4179	-0.0180
8	3	6	9	7	3	5	8	4450.5982	-0.0022
8	3	6	7	7	3	5	6	4450.5982	-0.0088
8	4	5	8	7	4	4	7	4451.0804	0.0012
9	1	9	-	8	1	8	-	4507.4581	-0.0041
8	3	5	8	7	3	4	7	4517.5393	-0.0067
8	3	5	9	7	3	4	8	4517.7165	-0.0018
8	3	5	7	7	3	4	6	4517.7165	-0.0090
9	0	9	-	8	0	8	-	4542.8651	0.0013
8	1	7	-	7	1	6	-	4557.9339	-0.0077

J'	K_a'	K_c'	F'	J''	K_a''	K_c''	F''	ν_{obs}	ν_{obs}-ν_{calc}
8	2	6	-	7	2	5	-	4672.7873	-0.0164
10	1	10	-	9	1	9	-	4989.4176	0.0032
9	3	7	9	8	3	6	8	5006.0321	-0.0001
9	3	7	8	8	3	6	7	5006.1394	-0.0084
9	3	7	10	8	3	6	9	5006.1394	-0.0089
10	0	10	-	9	0	9	-	5012.6587	-0.0063
9	4	6	9	8	4	5	8	5015.6881	0.0157
9	4	6	10	8	4	5	9	5015.9053	0.0288
9	4	6	8	8	4	5	7	5015.9053	0.0133
9	1	8	-	8	1	7	-	5069.5892	0.0117
9	3	6	8	8	3	5	7	5121.3437	-0.0089
9	3	6	10	8	3	5	9	5121.3437	-0.0085
9	2	7	-	8	2	6	-	5269.5456	-0.0119
10	2	9	-	9	2	8	-	5371.3226	0.0267
11	1	11	-	10	1	10	-	5469.3752	0.0004
11	0	11	-	10	0	10	-	5484.1455	0.0017
10	3	8	9	9	3	7	8	5557.7675	-0.0012
10	3	8	11	9	3	7	10	5557.7675	-0.0047
10	4	7	10	9	4	6	9	5581.8204	0.0148
10	4	7	11	9	4	6	10	5581.9337	-0.0222
10	4	7	9	9	4	6	8	5581.9337	-0.0292
10	3	7	9	9	3	6	8	5737.6086	0.0027
10	3	7	11	9	3	6	10	5737.6086	-0.0002
11	2	10	-	10	2	9	-	5876.7212	-0.0050
12	1	12	-	11	1	11	-	5947.9490	0.0157
12	0	12	-	11	0	11	-	5957.0750	-0.0066
11	1	10	-	10	1	9	-	6037.4484	0.0073
11	3	9	10	10	3	8	9	6104.1533	0.0187
11	3	9	12	10	3	8	11	6104.1533	0.0139
12	1	11	-	11	1	10	-	6502.8398	-0.0091
12	4	8	-	11	4	7	-	6781.7262	0.0123
13	2	12	-	12	2	11	-	6869.3338	-0.0153
14	1	14	-	13	1	13	-	6902.5311	-0.0136
14	0	14	-	13	0	13	-	6905.8713	0.0021
12	3	9	-	11	3	8	-	6987.8853	0.0040
13	3	11	-	12	3	10	-	7176.9131	0.0027
14	2	13	-	13	2	12	-	7358.1401	0.0011
15	1	15	-	14	1	14	-	7379.1549	-0.0019
15	0	15	-	14	0	14	-	7381.1201	-0.0004

J'	K_a'	K_c'	F'	J''	K_a''	K_c''	F''	ν_{obs}	$\nu_{obs}-\nu_{calc}$
13	4	9	-	12	4	8	-	7390.3629	-0.0159
14	1	13	-	13	1	12	-	7425.2787	0.0053
13	2	11	-	12	2	10	-	7495.8722	0.0021
13	3	10	-	12	3	9	-	7607.2746	0.0067
14	3	12	-	13	3	11	-	7701.9891	-0.0149
15	2	14	-	14	2	13	-	7843.1604	0.0212
16	1	16	-	15	1	15	-	7855.5420	0.0089
16	0	16	-	15	0	15	-	7856.6808	-0.0001
15	1	14	-	14	1	13	-	7888.9326	-0.0082
14	2	12	-	13	2	11	-	8000.3499	-0.0049
14	4	10	-	13	4	9	-	8012.6486	0.0012
14	3	11	-	13	3	10	-	8213.9729	-0.0060
15	3	13	-	14	3	12	-	8219.2600	0.0050
16	2	15	-	15	2	14	-	8325.2386	0.0009
17	1	17	-	16	1	16	-	8331.7710	0.0016
17	0	17	-	16	0	16	-	8332.4379	0.0034
16	1	15	-	15	1	14	-	8355.5365	-0.0041
15	2	13	-	14	2	12	-	8483.7872	0.0077
15	4	11	-	14	4	10	-	8646.5157	0.0091
16	3	14	-	15	3	13	-	8728.8710	-0.0030
15	3	12	-	14	3	11	-	8803.4869	-0.0136
17	2	16	-	16	2	15	-	8805.2108	-0.0009
18	1	18	-	17	1	17	-	8807.9307	0.0055
18	0	18	-	17	0	17	-	8808.3071	-0.0006
17	1	16	-	16	1	15	-	8824.7798	-0.0001
16	2	14	-	15	2	13	-	8950.5652	0.0004
16	4	13	-	15	4	12	-	8951.4929	0.0044
16	6	11	-	15	6	10	-	8954.5013	0.0018
16	5	11	-	15	5	10	-	9048.3019	-0.0181
17	3	15	-	16	3	14	-	9231.3836	-0.0070
18	2	17	-	17	2	16	-	9283.6894	-0.0044
19	1	19	-	18	1	18	-	9284.0322	-0.0042
19	0	19	-	18	0	18	-	9284.2552	0.0004
16	4	12	-	15	4	11	-	9286.7076	0.0204
18	1	17	-	17	1	16	-	9296.0853	-0.0007
16	3	13	-	15	3	12	-	9372.5706	-0.0043
17	2	15	-	16	2	14	-	9407.2122	0.0061
17	4	14	-	16	4	13	-	9496.0864	0.0040
17	6	11	-	16	6	10	-	9538.9971	-0.0191

J'	K_a'	K_c'	F'	J''	K_a''	K_c''	F''	ν_{obs}	ν_{obs}-ν_{calc}
17	5	13	-	16	5	12	-	9560.2585	0.0214
17	5	13	-	16	5	12	-	9560.2587	0.0216
17	5	12	-	16	5	11	-	9659.3456	-0.0064
18	3	16	-	17	3	15	-	9727.5917	0.0045
20	1	20	-	19	1	19	-	9760.1197	-0.0041
20	0	20	-	19	0	19	-	9760.2563	0.0085
19	2	18	-	18	2	17	-	9761.1725	0.0035
19	1	18	-	18	1	17	-	9768.8960	0.0038
18	2	16	-	17	2	15	-	9860.1959	0.0016
17	3	14	-	16	3	13	-	9918.6729	-0.0026
17	4	13	-	16	4	12	-	9925.5660	0.0161
18	4	15	-	17	4	14	-	10032.8433	-0.0003
19	3	17	-	18	3	16	-	10218.4036	-0.0037
21	1	21	-	20	1	20	-	10236.2130	0.0136
21	0	21	-	20	0	20	-	10236.2130	-0.0565
20	2	19	-	19	2	18	-	10237.9923	0.0023
20	1	19	-	19	1	18	-	10242.7376	-0.0017
19	2	17	-	18	2	16	-	10314.2186	-0.0085

Table S4. Cartesian coordinates (standard orientation) from the optimized structure of FA at the B3LYP/6-311++G(d,p) level of theory.

Atomic Number	Coordinates (Angstroms)		
	X	Y	Z
6	0.903243	0.000000	-0.000014
6	0.105738	1.170735	-0.000004
6	0.105739	-1.170735	-0.000005
6	-1.297116	0.712811	0.000011
6	-1.297115	-0.712810	0.000011
6	2.304425	0.000000	-0.000025
6	0.740447	2.394088	-0.000005
6	0.740446	-2.394088	-0.000007
6	-2.497647	1.412631	0.000022
6	-2.497647	-1.412631	0.000024
6	2.931239	1.277813	-0.000026
6	2.931238	-1.277812	-0.000027
6	2.162153	2.426643	-0.000018
6	2.162152	-2.426644	-0.000019
6	-3.699710	0.698031	0.000037
6	-3.699709	-0.698031	0.000037
1	0.186981	3.326893	0.000003
1	0.186980	-3.326893	0.000002
1	-2.509369	2.497250	0.000022
1	-2.509367	-2.497250	0.000024
1	4.013730	1.349945	-0.000035
1	4.013729	-1.349947	-0.000036
1	2.657076	3.391611	-0.000020
1	2.657078	-3.391609	-0.000020
1	-4.642046	1.234174	0.000047
1	-4.642046	-1.234174	0.000048

Table S5. Cartesian coordinates (standard orientation) from the optimized structure of FA at the MP2/6-311++G(d,p) level of theory.

Atomic	Coordinates (Angstroms)		
Number	X	Y	Z
6	0.904200	-0.000003	-0.001029
6	0.103466	1.170154	0.000516
6	0.103646	-1.170269	0.000342
6	-1.296526	0.714062	-0.000130
6	-1.296448	-0.714307	0.000206
6	2.313398	0.000100	0.000363
6	0.739796	2.403754	0.000045
6	0.740220	-2.403764	0.000113
6	-2.502683	1.420812	0.000022
6	-2.502643	-1.420973	-0.000004
6	2.939840	1.278912	-0.000467
6	2.940085	-1.278595	-0.000465
6	2.162692	2.435688	0.000369
6	2.163121	-2.435485	0.000229
6	-3.706840	0.702651	-0.000144
6	-3.706803	-0.702789	-0.000009
1	0.179673	3.336655	0.000496
1	0.180272	-3.336770	0.000542
1	-2.510959	2.508757	-0.000066
1	-2.510955	-2.508918	0.000055
1	4.025554	1.354983	-0.000317
1	4.025810	-1.354500	-0.000390
1	2.662215	3.401780	0.000157
1	2.662786	-3.401506	0.000058
1	-4.652788	1.238224	-0.000161
1	-4.652734	-1.238387	-0.000119

Table S6. Cartesian coordinates (standard orientation) from the optimized structure of 3-CNFA at the B3LYP/6-311++G(d,p) level of theory.

Atomic Number	Coordinates (Angstroms)		
	X	Y	Z
6	-1.937187	-2.040224	0.000076
6	-2.550060	-0.789753	0.000082
6	-1.751379	0.401330	0.000029
6	-0.367185	0.200765	-0.000050
6	0.263070	-1.069049	-0.000054
6	-0.530999	-2.196919	0.000011
1	-3.260371	1.970382	0.000123
1	-2.564831	-2.923256	0.000127
6	-2.199038	1.750527	0.000074
6	0.585634	1.248583	-0.000063
1	-0.111711	-3.196454	0.000030
6	0.128220	2.547814	-0.000004
6	-1.274831	2.778147	0.000057
1	0.806166	3.394119	0.000006
1	-1.630761	3.802239	0.000096
6	4.287036	0.248011	-0.000119
6	4.093186	-1.134297	-0.000099
6	2.803362	-1.674164	-0.000069
6	1.714264	-0.811342	-0.000062
6	1.911760	0.599573	-0.000086
6	3.197373	1.125150	-0.000114
1	5.295026	0.646929	-0.000139
1	4.951670	-1.796048	-0.000109
1	2.662946	-2.749535	-0.000065
1	3.361514	2.197089	-0.000130
6	-3.975173	-0.697800	0.000142
7	-5.128283	-0.609083	0.000222

Table S7. Cartesian coordinates (standard orientation) from the optimized structure of 3-CNFA at the MP2/6-311++G(d,p) level of theory.

Atomic Number	Coordinates (Angstroms)		
	X	Y	Z
6	1.934197	-2.060646	-0.000079
6	2.548409	-0.801383	-0.000083
6	1.757339	0.390469	-0.000044
6	0.364828	0.193095	0.000036
6	-0.272747	-1.073745	0.000026
6	0.523538	-2.211642	-0.000036
1	3.282077	1.952097	-0.000116
1	2.565966	-2.945536	-0.000115
6	2.215575	1.737299	-0.000072
6	-0.585327	1.245083	0.000041
1	0.096408	-3.212136	-0.000048
6	-0.117819	2.551740	-0.000001
6	1.288336	2.777014	-0.000047
1	-0.796937	3.401900	0.000002
1	1.651616	3.801815	-0.000074
6	-4.297641	0.267509	0.000133
6	-4.108290	-1.125031	0.000119
6	-2.818774	-1.675518	0.000090
6	-1.720218	-0.810836	0.000047
6	-1.911460	0.604361	0.000072
6	-3.201310	1.142144	0.000105
1	-5.306888	0.671071	0.000160
1	-4.973331	-1.783187	0.000142
1	-2.680869	-2.754626	0.000083
1	-3.357242	2.218804	0.000119
6	3.978112	-0.697413	-0.000130
7	5.151245	-0.580742	-0.000173

1. Experimental details and spectroscopic data

1.1. General synthetic methods

Reactions were carried out under argon using oven-dried glassware unless otherwise noted. DMF was purified by a MSBraun SPS-800 Solvent Purification System. Other commercial reagents were purchased from Sigma-Aldrich or BLD-Pharm and used without further purification. 3-bromofluoranthene was synthetized by an adapted published procedure.[1] Thin-layer chromatography (TLC) was performed on Merck silica gel 60 F_{254} and chromatograms were visualized with UV light (254 nm and 360 nm). Column chromatography was performed on Merck silica gel 60 (ASTM 40-60 µm). NMR spectra were recorded at 500 MHz and 125 MHz for ^{1}H and ^{13}C respectively (Bruker DRX-500). Gas chromatography/mass spectrometry (GC-MS) analysis were conducted on an Agilent 6890N instrument coupled to a HP-5973 Inert detector. Atmospheric pressure chemical ionization (APCI) HR spectra were obtained on a Bruker Microtof using Direct Inlet Probe (DIP) for sample introduction.

1.2. Experimental procedure for the synthesis of 3-cyanofluoranthene

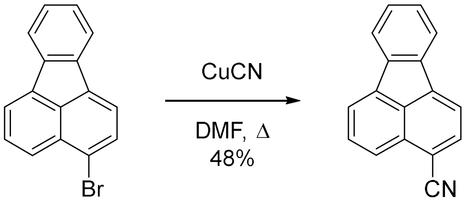

A mixture of 3-bromofluoroanthene (1.26 g, 4.48 mmol) and CuCN (1.63 g, 18.20 mmol) in dry DMF (50 mL) was heated to reflux for 16 h. The mixture was cooled to room temperature and then poured into water. The brownish solid was separated by filtration, washed with MeOH and further purified by column chromatography (SiO_2, hexane/CH_2Cl_2 9:1) to obtain 3-cyanofluoroanthene as a pale-yellow solid (486 mg, 2.14 mmol, 48%).

^{1}H NMR (500 MHz, $CDCl_3$) δ: 8.03 (d, J = 8.3 Hz, 1H), 8.00 (d, J = 7.2 Hz, 1H), 7.94 (d, J = 6.9 Hz, 1H), 7.90-7.86 (m, 3H), 7.74 (dd, J = 8.3, 7.0 Hz, 1H), 7.44 (td, J = 7.5, 1.3 Hz, 1H), 7.40 (td, J = 7.4, 1.2 Hz, 1H) ppm. **^{13}C RMN-DEPT** (126 MHz, $CDCl_3$) δ: 141.97 (C), 140.10 (C), 138.24 (C), 137.75 (C), 134.77 (CH), 132.10 (C), 130.60 (CH), 129.52 (CH), 128.43 (CH), 124.38 (CH), 122.79 (CH), 122.13 (CH), 121.71 (CH), 119.03 (CH), 117.79 (C), 108.63 (C) ppm.

---

[1] H. Hamamoto, D. Shimizu, K. Matsuda, *Chem. Eur. J.* **2024**, *30*, e202401353.

1.3. ^{1}H and ^{13}C NMR spectra

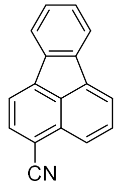

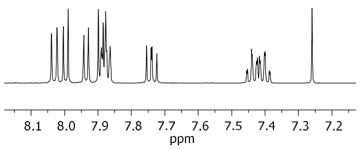

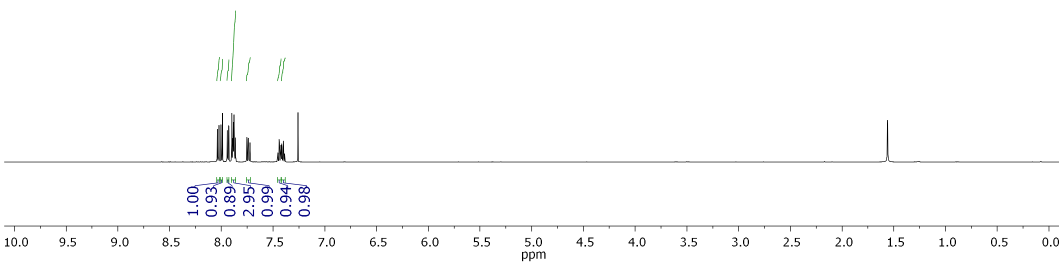

Figure S4: ^{1}H NMR spectrum of 3-cyanofluoranthene

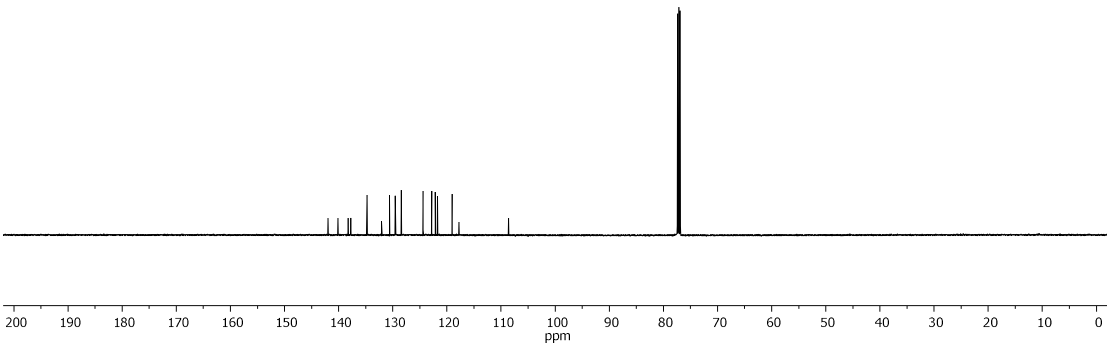

Figure S5: ^{13}C NMR (bottom) and DEPT-135 (top) spectra of 3-cyanofluoranthene
