# Supplementary material for: Rotational Characterization of Four-Ring Polycyclic Aromatic Hydrocarbons: Toward the Detection of Fluoranthene and Cyanofluoranthene in Space
Source: J Phys Chem Lett. 2026 Apr 17;17(17):5012–20. doi: 10.1021/acs.jpclett.6c00413 (PMC13137236; doi:10.1021/acs.jpclett.6c00413)
Supplement: Supplementary file 2 [file jz6c00413_si_002.pdf]

jz-2026-00413x.R1

Name: Peer Review Information for "Rotational Characterization of Four-ring Polycyclic Aromatic Hydrocarbons: Towards the detection of Fluoranthene and Cyanofluoranthene in Space"

First Round of Reviewer Comments

Reviewer: 1

Comments to the Author

Journal: The Journal of Physical Chemistry Letters

Manuscript ID: jz-2026-00413x

Title: Rotational Characterization of Four-ring Polycyclic Aromatic Hydrocarbons: Towards the detection of Fluoranthene and Cyanofluoranthene in Space

1. What is the major advance reported in the paper?

This study reports the first laboratory rotational spectroscopic characterization of the four-ring polycyclic aromatic hydrocarbon (PAH) fluoranthene and its synthesized derivative, 3-cyanofluoranthene. Using chirped-pulse Fourier-transform microwave spectroscopy supported by quantum chemical calculations, the authors have successfully determined the molecular parameters for these species.

2. What is the immediate significance of this advance?

The availability of accurate spectroscopic parameters provides the necessary rest frequencies to enable the search for these species in the interstellar medium. While the initial search in TMC-1 reported here resulted in a non-detection, the study establishes important upper limits for their abundance. The laboratory data provided are a prerequisite for future identification attempts in other astronomical sources, building on the recent detections of other PAHs and their cyano-derivatives.

3. Technical Suggestions

Main Text

Page 3, Line 38: Please add a citation to support the statement: “Most interstellar molecules, around 90% of those known, have been identified through their pure rotational spectrum via radio astronomy.”

Page 8, Line 18: Since both molecules are prolate rotors, could the authors explain the rationale for choosing the III-l representation rather than the I-r representation?

Page 8, Line 50: The definition of the inertial defect is slightly redundant. I suggest streamlining the sentence to: “…which are calculated from the determined principal moments of inertia according to Delta_c=Ic-Ib-Ia.”

Page 9, Top: Regarding the molecular planarity, what are the theoretical values of the inertia defects at the equilibrium positions? Be aware that the combination of MP2 theory with Pople basis sets is known to intrinsically produce artificial non-planar structures for planar aromatic systems (see DOI: 10.1021/ja0630285). Please ensure this artifact is not affecting your theoretical equilibrium values. Since anharmonic vibrational modes have already been calculated, please evaluate the specific contributions of the in-plane and out-of-plane modes to the vibrational ground state (see, for instance, DOI:10.1063/1.4905134).

Page 9, Lines 35-40: Please specify how the accuracy of the frequencies was estimated. Is it derived solely from the uncertainties of the fitted spectroscopic constants, or did the authors also consider other factors, such as the lack of information on higher-order centrifugal distortion constants?

Page 9, Lines 40-45: Please indicate the temperature used for the spectral simulations and the reasoning behind this choice. Additionally, clarify if the simulated spectra represent pure absorption.

Page 10, Lines 30-35: In addition to the reported physical parameters, please provide the coordinates of the astronomical source and the velocity (vLSR) used for the Doppler correction.

Page 10, Line 36: For context, it would be valuable to report the column density of other PAHs observed in the same source for comparison.

Page 12, Lines 20-25: I assume the VPT2 approach was used for the anharmonic calculations; please specify this explicitly in the text.

Figures and Tables

Figure 1: Please report the a and b principal inertial axes on the molecular structures.

Figure 3: Please specify the line profile type (Lorentzian, Gaussian, or Voigt) and the Full Width at Half Maximum (FWHM) used for the simulations.

Table 1: Given their small magnitude, the quartic centrifugal distortion constants should be reported in Hz rather than kHz. Please verify the values of the theoretical defects of inertia. A positive equilibrium value is physically impossible. This discrepancy might be due to the number of significant digits used in the rotational constants.

Supporting Information (SI)

Figure S1: Please number the atoms in the figure to facilitate the identification of isomers. It would also be useful to explicitly list the equivalent positions (e.g., 1-6, 2-5, 3-4, 7-10, 8-9).

Table S1: In the unit "MHz", the letter "z" is currently subscripted; please correct this typo. Units should be set in standard Roman (upright) font, not italics.

Table S3: Please check the assignment for the line at 2258.3988 MHz; the observed-calculated (o-c) value appears unusually large.

Tables S4–S5: Do the Cartesian coordinates refer to the principal axis system? Please also list the MP2 optimized structures.

Figure S4: Please explain the meaning of the top and bottom panels.

SI Page 13: Typo correction: 3-bromofluoroanthene -> 3-bromofluoranthene.

The manuscript is clearly written, concise, and logically organized. The experimental analysis is robust, and the results represent a valuable contribution to the field of laboratory astrophysics. I recommend the acceptance of this work in The Journal of Physical Chemistry Letters after the authors have addressed the revisions outlined above

Reviewer: 2

Comments to the Author

The authors report the first high-resolution rotational spectroscopic characterization of fluoranthene (FA) and its synthesized cyano derivative, 3-cyanofluoranthene (3-CNFA), using chirped-pulse Fourier-transform microwave spectroscopy. The laboratory measurements, supported by quantum chemical calculations, yield accurate rotational constants, centrifugal distortion constants, and nitrogen nuclear quadrupole coupling parameters. These spectroscopic data enable an astronomical search toward TMC-1 using

the QUIJOTE survey. Although 3-CNFA is not detected, a meaningful upper limit to its column density is derived.

The work is timely and relevant. Four-ring PAHs and their nitrile derivatives are currently at the forefront of astrochemical research, and laboratory spectral data remain essential for reliable astronomical searches. The study is carefully executed, methodologically sound, and appropriately cautious in its interpretation. The nondetection is reported transparently, and no overinterpretation is made.

Overall, the manuscript represents a valuable contribution to laboratory astrochemistry and is suitable for publication after minor revisions aimed at improving clarity, presentation, and methodological transparency.

1. Abstract: The abbreviation "3-CNFA" should be defined clearly at first occurrence in the Abstract or avoided there if unnecessary. The current formulation may be slightly abrupt for non-specialist readers.

2. In the text (p. 7), the authors state that the dipole moment component for 3-CNFA is 5.2 D. However, Table 1 and Table S1 report calculated values of μ_a = 5.1 D and μ_b = 0.2 D at the B3LYP level. Please ensure consistency between the text and tables (e.g., use 5.1 D throughout or clarify rounding). Besides, it would be useful to include the μ_c component in Table 1 (even if zero or negligible). This improves clarity and avoids ambiguity for readers less familiar with molecular symmetry arguments.

3. For FA, centrifugal distortion constants appear to have been set to zero, while for 3-CNFA certain constants (ΔK, δJ, δK) were fixed to theoretical values. The authors should clarify the rationale for this different treatment.

4. The derivation of the upper limit for 3-CNFA is an important result, yet the 3σ noise level is not explicitly indicated in Figure 4. In addition, the methodology used to derive the upper limit is only briefly mentioned. Please add a horizontal dashed line indicating the 3σ detection threshold in each panel of Figure 4. Provide a short explanation in the text describing how the upper limit was calculated (e.g., assumed linewidth, excitation temperature, source size, stacking procedure).

5. The colored stars (green, red, blue) in Figure 4 indicating detected, nondetected, and blended transitions are not explained in the figure caption. The caption should be self-contained. In addition, for features marked as coincident with predicted frequencies but not attributed to 3-CNFA, the authors should clarify whether these are fully assigned to known species, partially blended, or unassigned lines.

6. The concluding statement currently reads: “This study highlights the increasing capability of chirped-pulse broadband microwave spectroscopy ... to identify new aromatic molecules in space.” Since no new astronomical detection is reported here, a slightly more measured formulation would be appropriate. For example: “... to support ongoing and future astronomical searches for aromatic molecules in space.” This adjustment would better reflect the scope of the present work.

7. Please revise the reference formatting to conform strictly to the journal style (e.g., ensure journal titles are present and correctly abbreviated; for example, Reference 1 appears incomplete).

Author's Response to Peer Review Comments:

13 March 2026

Dear Editor,

Thank you for your email with the reviewers’ report on our manuscript titled **“Rotational Characterization of Four-ring Polycyclic Aromatic Hydrocarbons: Towards the detection of Fluoranthene and Cyanofluoranthene in Space”** by Daniel Villar-Castro, Carlos Cabezas, Amanda L. Steber, José R. Morán, Selene de la Fuente, Farha. S. Hussain, Dolores Pérez, Alberto Lesarri, José Cernicharo, Cristóbal Pérez and myself. We thank the reviewers for their valuable comments and suggestions. Their feedback helped us improve the manuscript. Our point-by-point responses are below. The changes in the manuscript have been highlighted in yellow.

Reviewer #1

1. What is the major advance reported in the paper?

This study reports the first laboratory rotational spectroscopic characterization of the four-ring polycyclic aromatic hydrocarbon (PAH) fluoranthene and its synthesized derivative, 3cyanofluoranthene. Using chirped-pulse Fourier-transform microwave spectroscopy supported by quantum chemical calculations, the authors have successfully determined the molecular parameters for these species.

2. What is the immediate significance of this advance?

The availability of accurate spectroscopic parameters provides the necessary rest frequencies to enable the search for these species in the interstellar medium. While the

initial search in TMC-1 reported here resulted in a non-detection, the study establishes important upper limits for their abundance. The laboratory data provided are a prerequisite for future identification attempts in other astronomical sources, building on the recent detections of other PAHs and their cyano-derivatives. 3. Technical Suggestions

Main Text

Page 3, Line 38: Please add a citation to support the statement: "Most interstellar molecules, around 90% of those known, have been identified through their pure rotational spectrum via radio astronomy." **It has been added.**

Page 8, Line 18: Since both molecules are prolate rotors, could the authors explain the rationale for choosing the III-l representation rather than the I-r representation?

The III-l representation was chosen because it allowed us to determine two quartic centrifugal distortion constants, whereas the I-r representation yielded only one.

Page 8, Line 50: The definition of the inertial defect is slightly redundant. I suggest streamlining the sentence to: "...which are calculated from the determined principal moments of inertia according to Delta_c=Ic-Ib-Ia."

It has been changed in the text.

Page 9, Top: Regarding the molecular planarity, what are the theoretical values of the inertia defects at the equilibrium positions? Be aware that the combination of MP2 theory with Pople basis sets is known to intrinsically produce artificial non-planar structures for planar aromatic systems (see DOI: 10.1021/ja0630285). Please ensure this artifact is not affecting your theoretical equilibrium values. Since anharmonic vibrational modes have already been calculated, please evaluate the specific contributions of the in-plane and out-of-plane modes to the vibrational ground state (see, for instance, DOI:10.1063/1.4905134).

Theoretical values of the inertial defects at the equilibrium positions at the B3LYP/6-311++G(d,p) level of theory indicated in Table 1 have been corrected and set to zero. MP2 theory has not been used to calculate inertia defects. A sentence has been added to include the in-plane and out-of-plane modes to the vibrational ground state: "…To confirm this, anharmonic vibrational modes were calculated at the B3LYP/6-311++G(d,p) level of theory. The results show that the three lowest vibrational modes of FA, predicted at 100, 120, and 163 cm^{-1}, correspond to OOP motions. Similar results were obtained for 3-CNFA: the two lowest vibrational modes, at 62 and 116 cm^{-1}, are also OOP motions, whereas the mode at 121 cm^{-1} corresponds to the first in-plane vibration, with the main contribution arising from the nitrile group…"

Page 9, Lines 35-40: Please specify how the accuracy of the frequencies was estimated. Is it derived solely from the uncertainties of the fitted spectroscopic constants, or did the

authors also consider other factors, such as the lack of information on higher-order centrifugal distortion constants?

The frequencies were estimated from the rotational constants derived in the laboratory. Considering the rigidity of the molecule, we expect that the extrapolation from the frequency range covered in the laboratory to the K band (18–26 GHz) is accurate. Including unmeasured (theoretical) constants would introduce additional uncertainties in the predictions. A sentence has been added to clarify the origin of the quoted uncertainties.

Page 9, Lines 40-45: Please indicate the temperature used for the spectral simulations and the reasoning behind this choice. Additionally, clarify if the simulated spectra represent pure absorption. **We have assumed that the rotational temperature is 8 K as already mentioned in the text and several references are provided to justify this choice. We would like to note that for such large molecules the collision rates could be large. We have studied in detail the case of benzonitrile (C_6H_5CN Cernicharo et al. 2021, A&A, 655, L1; see their appendix C) for which we derive a Trot close to 9 K. The expected signals are all in emission. Only a few molecules show anomalous absorption against cosmic background radiation.**

Page 10, Lines 30-35: In addition to the reported physical parameters, please provide the coordinates of the astronomical source and the velocity (vLSR) used for the Doppler correction. **Position and v_{LSR} have been included in page 9.**

Page 10, Line 36: For context, it would be valuable to report the column density of other PAHs observed in the same source for comparison.

The purpose of the paper is to search for cyanofluoranthene. We have added a sentence concerning the abundance of similar PAHs; however, a discussion of the implications of the different column densities would require detailed chemical modeling, which is beyond the scope of this paper.

Page 12, Lines 20-25: I assume the VPT2 approach was used for the anharmonic calculations; please specify this explicitly in the text.

We prefer not to specify the VPT2 approach in the text.

Figures and Tables

Figure 1: Please report the a and b principal inertial axes on the molecular structures. **The principal inertial axes have been included in Figure S3 of SI.**

Figure 3: Please specify the line profile type (Lorentzian, Gaussian, or Voigt) and the Full Width at Half Maximum (FWHM) used for the simulations.

It has been specified in the legend of Figure 3: "…A Gaussian line profile and a typical full width at half-maximum (FWHM) linewidth of ca. 100 kHz were used for the simulation…"

Table 1: Given their small magnitude, the quartic centrifugal distortion constants should be reported in Hz rather than kHz. Please verify the values of the theoretical defects of inertia. A positive equilibrium value is physically impossible. This discrepancy might be due to the number of significant digits used in the rotational constants.

We prefer to maintain units in kHz. The theoretical values of the inertia defect are zero when we take more digits in the rotational constants; these values have been corrected in Table 1.

Supporting Information (SI)

Figure S1: Please number the atoms in the figure to facilitate the identification of isomers. It would also be useful to explicitly list the equivalent positions (e.g., 1-6, 2-5, 3-4, 7-10, 8-9).

Numbering of the atoms have been included in figure S1, and the list of the equivalent positions has been detailed in Figure S1: "Schematic structure of FA (central) and its cyano derivatives (CNFA) optimized at the B3LYP-6-311++G(d,p) level of theory. The atom numbering of FA indicates the possible nitrile substitution sites corresponding to the five CNFA isomers. The following positions are symmetry-equivalent: 1–6, 2–5, 3–4, 7–10, and 8–9".

Table S1: In the unit "MHz", the letter "z" is currently subscripted; please correct this typo. Units should be set in standard Roman (upright) font, not italics. **Corrected**

Table S3: Please check the assignment for the line at 2258.3988 MHz; the observed-calculated (o-c) value appears unusually large.

The transition 422–321 (quadrupole component F = 3–2) was assigned to two frequencies; the one at 2258.3988 MHz was incorrect. The new molecular parameters obtained for 3-CNFA have been changed in Table 1, as well as the list of frequencies in Table S3 of Supporting Information.

Tables S4–S5: Do the Cartesian coordinates refer to the principal axis system? Please also list the MP2 optimized structures.

No, they are not referred to the principal axis system. The text has been changed as follows: "…Cartesian coordinates (standard orientation) from the optimized structure…" Furthermore, MP2 optimized structures (Tables S5 and S7) have been added to the SI.

Figure S4: Please explain the meaning of the top and bottom panels.

Figure S4 is now Figure S5. The lower part of the figure shows the ^{13}C NMR spectrum of the compound, which displays the signals of all carbon atoms. The upper part shows the DEPT-135 spectrum (DEPT = Distortionless Enhancement by Polarization Transfer), a commonly used NMR experiment that registers only the signals corresponding to carbon atoms bonded to hydrogen and removes signals from quaternary carbons. The Figure S4 caption has been modified to clarify this point: ^{13}C NMR (bottom) and DEPT-135 (top) spectra of 3-cyanofluoranthene

SI Page 13: Typo correction: 3-bromofluoroanthene -> 3-bromofluoranthene.
Corrected

The manuscript is clearly written, concise, and logically organized. The experimental analysis is robust, and the results represent a valuable contribution to the field of laboratory astrophysics. I recommend the acceptance of this work in The Journal of Physical Chemistry Letters after the authors have addressed the revisions outlined above

Reviewer #2

The authors report the first high-resolution rotational spectroscopic characterization of fluoranthene (FA) and its synthesized cyano derivative, 3-cyanofluoranthene (3-CNFA), using chirped-pulse Fouriertransform microwave spectroscopy. The laboratory measurements, supported by quantum chemical calculations, yield accurate rotational constants, centrifugal distortion constants, and nitrogen nuclear quadrupole coupling parameters. These spectroscopic data enable an astronomical search toward TMC-1 using the QUIJOTE survey. Although 3-CNFA is not detected, a meaningful upper limit to its column density is derived.

The work is timely and relevant. Four-ring PAHs and their nitrile derivatives are currently at the forefront of astrochemical research, and laboratory spectral data remain essential for reliable astronomical searches. The study is carefully executed, methodologically sound, and appropriately cautious in its interpretation. The nondetection is reported transparently, and no overinterpretation is made.

Overall, the manuscript represents a valuable contribution to laboratory astrochemistry and is suitable for publication after minor revisions aimed at improving clarity, presentation, and methodological transparency.

1. Abstract: The abbreviation "3-CNFA" should be defined clearly at first occurrence in the Abstract or avoided there if unnecessary. The current formulation may be slightly abrupt for non-specialist readers.
It has been defined at first occurrence in the abstract. The text has been changed as follows: "…In this work, we report a rotational spectroscopic study of commercially available fluoranthene and its synthesized cyano derivative, 3-cyanofluoranthene (3-CNFA), using…"

2. In the text (p. 7), the authors state that the dipole moment component for 3-CNFA is 5.2 D. However, Table 1 and Table S1 report calculated values of μ_a = 5.1 D and μ_b = 0.2 D at the B3LYP level. Please ensure consistency between the text and tables (e.g., use 5.1 D throughout or clarify rounding). Besides, it would be useful to include the µ_c component in Table 1 (even if zero or negligible). This improves clarity and avoids ambiguity for readers less familiar with molecular symmetry arguments. **We have ensured consistency between the text and tables using a value of μ_a = 5.1 D, and the μ_c component has been added in Table 1.**

3. For FA, centrifugal distortion constants appear to have been set to zero, while for 3-CNFA certain constants (ΔK, δJ, δK) were fixed to theoretical values. The authors should clarify the rationale for this different treatment.
Centrifugal distortion constants could not be determined for FA; therefore, the rigid rotor model was applied. For 3-CNFA, two centrifugal distortion constants were successfully determined, while the remaining three were fixed to their theoretical values to improve the accuracy of spectral predictions at higher frequencies for astronomical searches.

4. The derivation of the upper limit for 3-CNFA is an important result, yet the 3σ noise level is not explicitly indicated in Figure 4. In addition, the methodology used to derive the upper limit is only briefly mentioned. Please add a horizontal dashed line indicating the 3σ detection threshold in each panel of Figure 4. Provide a short explanation in the text describing how the upper limit was calculated (e.g., assumed linewidth, excitation temperature, source size, stacking procedure).
The figure has been modified following the referee's suggestion. The assumed linewidth, excitation temperature and source size were already provided in text.

5. The colored stars (green, red, blue) in Figure 4 indicating detected, nondetected, and blended transitions are not explained in the figure caption. The caption should be self-contained. In addition, for features marked as coincident with predicted frequencies but not attributed to 3-CNFA, the authors should clarify whether these are fully assigned to known species, partially blended, or unassigned lines. **We have added the corresponding sentences to explain the meaning of the stars. None of the features that appear associated with predicted transitions are above the 3sigma level. Lines above 3sigma that can be assigned to known species are indicated.**

6. The concluding statement currently reads: "This study highlights the increasing capability of chirped-pulse broadband microwave spectroscopy ... to identify new aromatic molecules in space." Since no new astronomical detection is reported here, a slightly more measured formulation would be appropriate. For example: "... to support ongoing and future astronomical searches for aromatic molecules in space." This adjustment would better reflect the scope of the present work.
The text has been revised in accordance with the reviewer's comments: "…This study highlights the increasing capability of chirped-pulse broadband microwave spectroscopy, combined with targeted astronomical observations, to support ongoing and future astronomical searches for aromatic molecules in space…"

7. Please revise the reference formatting to conform strictly to the journal style (e.g., ensure journal titles are present and correctly abbreviated; for example, Reference 1 appears incomplete). **We have updated some references.**

Yours faithfully,

Isabel Peña, on behalf of all authors
